# Entry and exit of chemotherapeutically-promoted cellular dormancy in glioblastoma cells is differentially affected by the chemokines CXCL12, CXCL16, and CX3CL1

**DOI:** 10.1038/s41388-020-1302-8

**Published:** 2020-04-28

**Authors:** Vivian Adamski, Kirsten Hattermann, Carolin Kubelt, Gesa Cohrs, Ralph Lucius, Michael Synowitz, Susanne Sebens, Janka Held-Feindt

**Affiliations:** 1 Department of Neurosurgery, University Medical Center Schleswig-Holstein UKSH, Campus Kiel, 24105 Kiel, Germany; 2 Department of Anatomy, University of Kiel, 24118 Kiel, Germany; 3 Institute for Experimental Cancer Research, University of Kiel and University Medical Center Schleswig-Holstein UKSH, Campus Kiel, 24105 Kiel, Germany

**Keywords:** Chemotherapy, CNS cancer

## Abstract

*Glioblastoma multiforme* (GBM) is a malignant brain tumor that evades therapy regimens. Since cellular dormancy is one strategy for surviving, and since chemokines determine the environmental conditions in which dormancy occurs, we investigated how chemokines affect temozolomide (TMZ)-promoted cellular dormancy entry and exit in GBM cells. TMZ administration over ten days promoted cellular dormancy entry, whereas discontinuing TMZ for a further 15 days resulted in resumption of proliferation. Co-administration of a chemokine cocktail containing CXCL12, CXCL16, and CX3CL1 resulted in both delayed entry and exit from cellular dormancy. A microarray-based transcriptome analysis in LN229 GBM cells revealed that cellular dormancy entry was characterized by an increased expression of CCL2 and SAA2, while THSD4, FSTL3, and VEGFC were upregulated during dormancy exit. Co-stimulation with the chemokine cocktail reduced upregulation of identified genes. After verifying the appearance of identified genes in human GBM primary cultures and ex vivo samples, we clarified whether each chemokine alone impacts cellular dormancy mechanisms using specific antagonists and selective CRISPR/Cas9 clones. While expression of CCL2 and SAA2 in LN229 cells was altered by the CXCL12-CXCR4-CXCR7 axis, CXCL16 and CX3CL1 contributed to reduced upregulation of THSD4 and, to a weaker extent, of VEGFC. The influence on FSTL3 expression depended on the entire chemokine cocktail. Effects of chemokines on dormancy entry and exit-associated genes were detectable in human GBM primary cells, too, even if in a more complex, cell-specific manner. Thus, chemokines play a significant role in the regulation of TMZ-promoted cellular dormancy in GBMs.

## Introduction

*Glioblastoma multiforme* (GBM) is a disease with a poor prognosis due to resistance to chemotherapy and radiotherapy [1]. Evolutionary processes within the heterogeneous tumor mass give rise to specialized tumor cell subpopulations [2–6], which adapt to their microenvironment and manage to survive therapeutic strategies.

One strategy by which tumor cells escape treatment effects is entering a dormant state which might occur via two mechanisms: tumor mass dormancy and cellular dormancy. In tumor mass dormancy tumors remain occult, do not expand in size for a long time, which might also occur in minimal residual disease after surgical removal or treatment of the tumor [7–13]. In tumor mass dormancy, there is a balance of proliferating and dying tumor cells which is achieved by and dependent on immune cells in the direct proximity (immunesurveillance) or an insufficient angiogenic potential. In contrast, during cellular dormancy solitary tumor cells undergo a temporary quiescence which is based on a growth arrest which can be promoted, e.g., by chemotherapy [7–13].

The existence of dormancy was proven in GBMs [10–17] and is characterized by the upregulation of a specific dormancy-associated gene set [17]. Dormancy contributes to a poor therapy outcome in GBMs [18], and the occurrence of a therapy-driven plasticity of GBM cells towards a predominantly drug-promoted cellular dormant phenotyp*e* in vitro results in cell-type specific responses to chemotherapy-mediated cytotoxicity [19].

The evolution of individual cell subpopulations in the GBM ecosystem takes place under the pressure of microenvironmental factors. Here, among others, chemokines determine the distinct, inflammatory environmental conditions.

Chemokines and their receptors play a decisive role in tumor progression. They regulate tumor growth either directly by impacting transformation, survival, proliferation and migration of cancer cells, or indirectly by enhancing angiogenesis or recruiting leukocytes [20–24]. In GBMs, they affect tumor progression in a multi-faceted way. For example, CXCL12 (SDF-1, stromal cell-derived factor-1) mediates proliferative, migratory or anti-apoptotic effects via its receptors CXCR4 and CXCR7 [25–28]. The transmembrane chemokines CXCL16 and CX3CL1 promote pro-tumorigenic effects via classical and alternative signaling pathways [29–33]. Thus, a complex chemokine-signaling network is involved in glioma progression.

However, it is still unknown whether chemokines affect drug-promoted cellular dormancy in GBMs. Thus, we studied TMZ-promoted cellular dormancy entry and exit in human GBM cells and investigated the impact of defined chemokines on this important tumor biological phenomenon.

## Results

### TMZ-treated LN229 GBM cells are a reliable in vitro model for investigating cellular dormancy entry and exit and the influence of chemokines on these processes

**Fig. 1:**
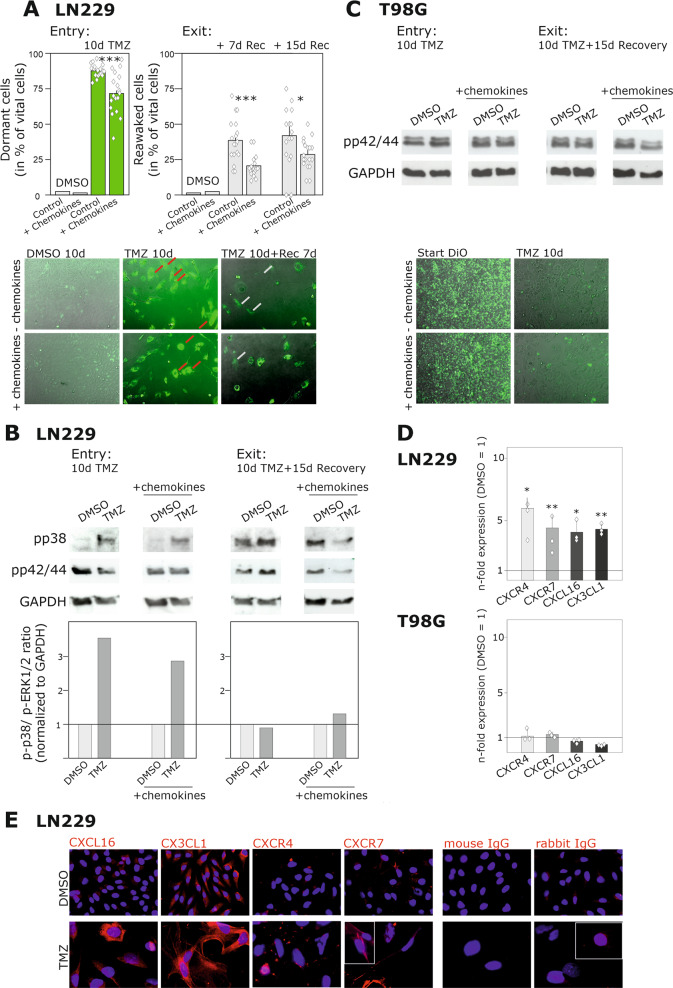
TMZ-treated GBM cells as a reliable in vitro model to investigate cellular dormancy entry and exit, and the influence of chemokines on these processes. To investigate TMZ-promoted cellular dormancy entry and exit mechanisms and a possible influence of chemokines on these processes, partially drug-sensitive LN229 cells and drug-resistant T98G cells were stimulated with 500 µM TMZ or 0.5% (v/v) DMSO, respectively, with or without a chemokine mix containing 2 nM CXCL12, CXCL16, and CX3CL1 for ten days. The cells were incubated for a further 15 days without TMZ but with or without the chemokine mix. To confirm the results by numbers, stimulated GBM cells were stained with DiO, and the ratio of dye retaining cells to total vital cell count was calculated (*n* = 2 biological replicates with *n* = 8–9 technical replicates, respectively) after ten days of chemotherapeutic treatment, as well as after seven and 15 days without this stimulus (**a** and **c**, magnification ×100). In addition, signaling via the p42/44 and p38 MAPK pathways was analyzed by western blot after ten days of stimulation with TMZ and with or without the chemokine mix, and after a further 15 days of recovery (**b** and **c**; exemplary data shown; *n* = 4 biological replicates with *n* = 1 technical replicate, respectively). The regulation of the gene and protein expression of the respective chemokine receptors and ligands was investigated by qRT-PCR and immunocytochemistry, respectively, after ten days of TMZ-treatment (**d** and **e**; *n* = 3 biological replicates with *n* = 2 technical replicates, respectively; magnification ×200). Statistical analysis of the cell number differences in drug-promoted cellular dormancy entry was performed by unpaired, two-sided Student’s *t*-test, whereas those in cellular dormancy exit were analyzed by repeated two-way ANOVA with Bonferroni’s multiple comparison post hoc test. Gene regulation after TMZ stimulation was statistically analyzed by paired, two-sided Student’s *t*-test. *p < 0.05, **p < 0.01 and ***p < 0.001. DMSO, dimethyl sulfoxide; GAPDH, glycerinaldehyde 3-phosphate dehydrogenase; Rec: recovery; TMZ, temozolomide.

In accordance with our previous results [19], we were able to induce drug-promoted cellular dormancy entry in LN229 cells after ten days of TMZ-application. LN229 cells are known to be partially TMZ-sensitive, probably due to a low O^6^-methylguanine-DNA methyltransferase (MGMT) expression [34, 35]. TMZ is a common GBM chemotherapeutic which, besides other mechanisms, is able to induce cellular quiescence by promoting cell cycle-arrest [36]. Indeed, as previously shown by cytotoxicity analysis [19], most LN229 cells died during a continuous ten-day TMZ-stimulation, however, some cells survived this treatment. These cells mainly exhibited an enlarged morphology with large nuclei [19] and were also characterized by DiO-retention and larger intracellular phospho-p38 amounts in relation to phospho-p42/44 signals (Fig. 1a, b), as shown previously [19] and in line with dormancy criteria described in the literature [37, 38]. In addition, TMZ-treated LN229 cells were characterized by a negative staining for the proliferation marker Ki-67 (Supplementary Fig. 1). Altogether, these results clearly indicate the acquisition of a cellular dormant phenotype in TMZ-treated surviving LN229 cells.

After 15 days of TMZ-withdrawal, we observed a recovery of the LN229 cells, evidenced by the successive appearance of DiO-negative, as well as the occurrence of a phospho-p38/phospho-p42/44 ratio similar to DMSO controls. Overall, these findings strongly support the view that LN229 cells undergo a TMZ-promoted cellular dormant stage which is reversible after TMZ-withdrawal (Fig. 1a, b). Interestingly, applying the chemokine cocktail during TMZ-treatment and the TMZ-free recovery period reduced both cellular dormancy entry and exit. The numbers of DiO-positive (dormancy entry), as well as DiO-negative (dormancy exit) LN229 cells were lower than in experiments without chemokines. Furthermore, the phospho-p38/phospho-p42/44 ratios decreased during cellular dormancy entry and were slightly increased during cellular dormancy exit compared with respective experiments without chemokines. Both mRNA and protein expression of CXCR4 and CXCR7, known chemokine receptors mediating CXCL12 signals, as well as of the chemokine ligands CXCL16 and CX3CL1 were induced in LN229 cells during TMZ-treatment (Fig. 1d, e). The chemokine receptors CXCR6 and CX3CR1, which are usually known to mediate CXCL16 and CX3CL1 signals, were not expressed in the LN229 cells (data not shown). However, via a recently published mechanism known as “inverse signaling” [32], soluble CXCL16 and CX3CL1 molecules are able to bind to their transmembrane counterparts resulting in the induction of intracellular signal cascades and cellular responses.

In comparison, neither induction of cellular quiescence nor regulation of intracellular signal cascades, or CXCR4, CXCR7, CXCL16, or CX3CL1 expression were altered in TMZ-treated T98G cells (Fig. 1c, d). These results are in accordance with previously published data, which show that T98G cells are characterized by a pronounced TMZ resistance, probably due to a strong MGMT expression [34, 35]. However, it should be clearly stated that T98G cells cannot actually be called “non-dormant” since they are able to induce dormant tumors in vivo with proliferation potentials similar to native, non-drug-treated T98G cells in vitro [12, 17]. Since we particularly aim to investigate the impact of chemokines on entry and exit of TMZ-promoted cellular dormancy, it should be kept in mind that the results of the different cell lines will compared only with regard to this specific aspect.

### Chemokines affect several genes associated with TMZ-promoted cellular dormancy entry and exit

**Fig. 2:**
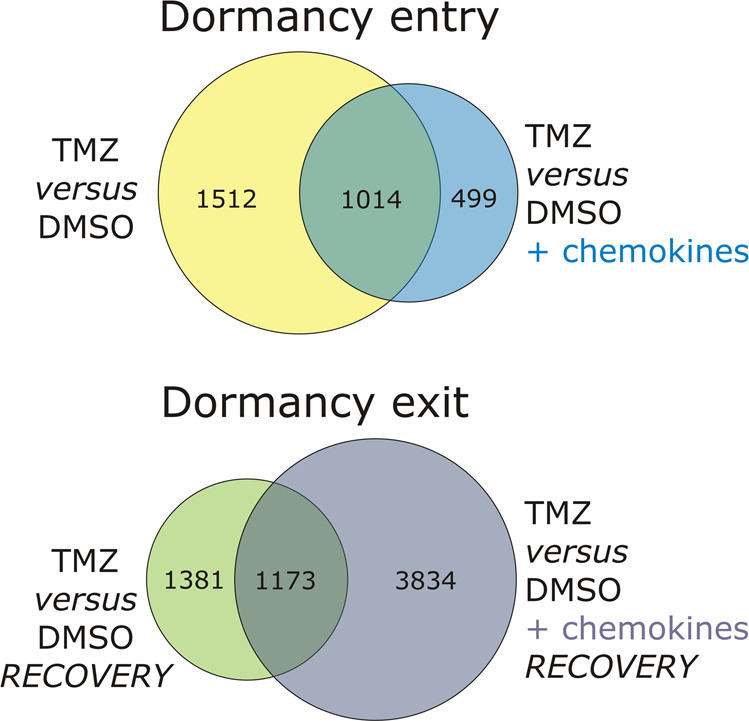
Microarray-based transcriptome analysis of the influence of chemokines on drug-promoted cellular dormancy entry and exit in LN229 GBM cells. Cellular dormancy entry was promoted by stimulating with 500 µM TMZ or 0.5% (v/v) DMSO, respectively, with or without a chemokine mix containing 2 nM CXCL12, CXCL16, and CX3CL1 for ten days. The cells were incubated for a further 15 days without TMZ but with or without the chemokine mix (*n* = 3 biological replicates). Transcriptome analysis was performed using the ArrayXS® human 8 × 60 K microarray, and n-fold expression differences were displayed as log2-fold changes (log2FC) with a log2FC = 2 value indicating a 4-fold expression difference. All log2FC values between −1 and 1 were excluded, and only statistically significant values were enclosed. Venn diagrams for cellular dormancy entry (top) and exit (bottom) were prepared with each circle representing genes that had been identified as being significantly differentially expressed after TMZ-stimulation versus DMSO control, as well as in the TMZ versus DMSO + chemokines groups, respectively. Areas of overlap represent genes that are significantly differentially expressed in both groups. The numbers in the Venn diagrams indicate the number of genes in the particular Venn area. Complete gene lists including log2FC and *p* values of different Venn areas are given in Supplementary Tables 1 and 2.

To analyze the influence of chemokines on LN229 gene expression during drug-promoted cellular dormancy entry and exit, a microarray-based transcriptome analysis was performed using an ArrayXS human 8 × 60 K microarray, and Venn diagrams were prepared for cellular dormancy entry and exit (Fig. 2). Complete gene lists including log2FC and *p*-values of different Venn areas are given in Supplementary Tables 1 and 2.

1512 genes were regulated in LN229 cells during TMZ-promoted cellular dormancy entry. 499 genes were regulated to significant extents when the cellular dormant state was induced by TMZ with additional chemokine application, and 1014 genes were regulated in both groups. Regarding cellular dormancy exit, 1381 genes were differentially expressed in the TMZ versus DMSO recovery group, 3834 in the TMZ versus DMSO plus chemokines recovery group, and 1173 were regulated in both groups. To narrow down the number of interesting genes, we defined three conditions: 1. genes have to be regulated during TMZ-promoted cellular dormancy entry or exit (1512 and 1381 gene groups, respectively) with at least a log2FC = 2 value; 2. when comparing log2FC values of TMZ-promoted cellular dormancy entry-associated or exit-associated genes with those values resulting after additional chemokine application, chemokines should regulate the distinct genes or not all (dormancy entry: 1014 and/or 1512 gene groups; dormancy exit: 1173 and/or 1381 gene groups); and 3. only known genes were considered that were expressed to clearly detectable extents after TMZ-treatment and that could also be analyzed at the protein level. According to these restrictions, we decided to exemplarily investigate the genes *CCL2* and *SAA2* coding for the CC-chemokine ligand 2 and the serum amyloid A protein 2 for cellular dormancy entry, and the genes *FSTL3, VEGFC*, and *THSD4* coding for the follistatin-related protein 3, the thrombospondin type 1 domain containing 4 protein, and the vascular endothelial growth factor C for cellular dormancy exit.

### Validation of the significance of TMZ-promoted cellular dormancy entry-associated and exit-associated genes and impact of chemokines

**Fig. 3:**
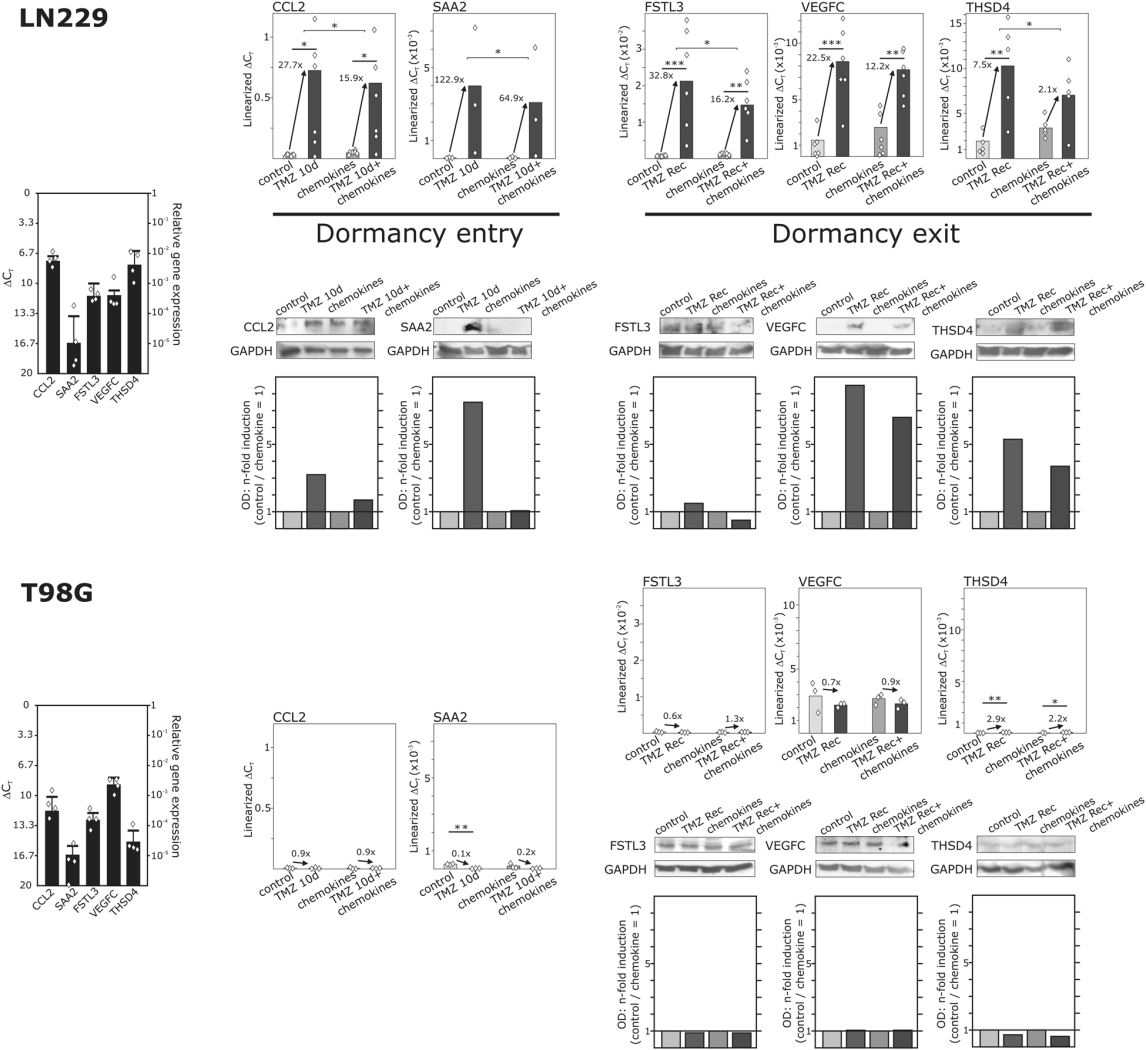
Validation of the influence of chemokines on TMZ-promoted cellular dormancy entry-associated and exit-associated gene expression. Basic gene expression levels were detected in LN229 (partially TMZ-sensitive) and T98G cells (TMZ-resistant) by qRT-PCR (left diagrams; *n* = 4 biological replicates with *n* = 2 technical replicates, respectively). LN229 (top) and T98G cells (bottom) were treated with 500 µM TMZ or 0.5% (v/v) DMSO, respectively, with or without a chemokine mix containing 2 nM CXCL12, CXCL16, and CX3CL1 for ten days followed by 15 days without TMZ but with chemokine stimulation. Regulation of identified cellular dormancy entry-associated and exit-associated genes was determined after ten and 25 days of stimulation by qRT-PCR and western blot (for qRT-PCT exact numbers of biological replicates were shown in the figure with *n* = 2 technical replicates; for western blot *n* = 2 biological replicates with *n* = 1 technical replicate; OD = optical density; exemplary data shown). Gene regulation after TMZ-stimulation was statistically analyzed by repeated two-way ANOVA with Bonferroni’s multiple comparison post hoc test. The effects with and without chemokines were analyzed by paired, two-sided Student’s *t*-test. *p < 0.05, **p < 0.01, and ***p < 0.001.

To confirm the results of the transcriptome analysis, we used our above described GBM in vitro model and analyzed the data both at the mRNA and protein level. CCL2 and SAA2 were indeed expressed in LN229 cells (Fig. 3 top, left diagram), and their mRNA and protein expression was clearly induced during TMZ-promoted cellular dormancy entry, whereas this induction was not as prominent when cellular dormancy entry was induced with TMZ and additional chemokine stimulation (Fig. 3 top). Although T98G cells did not respond to TMZ-treatment with induction of a cellular dormant state, basal CCL2 and SAA2 expression was also confirmed in these cells (Fig. 3 bottom, left diagram). However, in these cells no TMZ-promoted regulation of CCL2 and SAA2 expression was detectable, and no influence of chemokines on CCL2 and SAA2 expression was observed (Fig. 3 bottom). Protein signals were below the detection level in T98G cells due to the absent TMZ-promoted induction of CCL2 and SAA2 expression.

When investigating the cellular dormancy exit, both mRNA expression and protein synthesis of cellular dormancy exit-associated molecules FSTL3, THSD4, and VEGFC were clearly induced in TMZ-treated LN229 cells, and this induction was diminished by additional chemokine application (Fig. 3 top). FSTL3, THSD4, and VEGFC were detected both at the mRNA and the protein level in T98G, as well. However, no convincing neither TMZ-promoted nor chemokine-induced regulation of these genes and proteins was observed (Fig. 3 bottom).

**Fig. 4:**
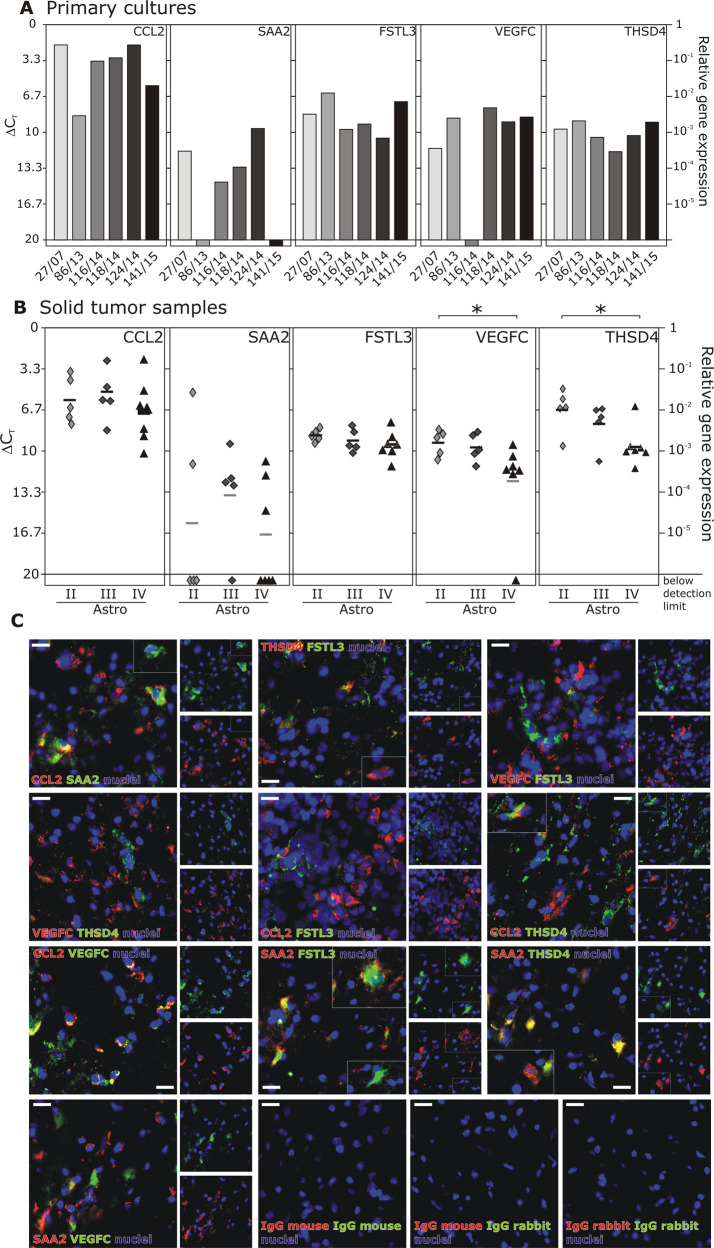
Cellular dormancy entry-associated and exit-associated genes are expressed in human GBM primary cultures (six different patients), solid glioma samples of various malignancy grades (Astro II; *n* = 5, Astro III; *n* = 5 different patients, respectively), and ex vivo GBM samples (*n* = 7 different patients). Primary cultures of different human GBMs (**a**), as well as solid glioma samples of differing malignancy grades (**b**) were analyzed by qRT-PCR with regard to the expression of cellular dormancy entry-associated and exit-associated genes (*n* = 2 technical replicates for each biological different primary culture or solid tumor sample). **c** Human GBM sections (*n* = 3 different patients) were immunofluorescently stained ex vivo regarding the presence of and co-staining for CCL2, SAA2, FSTL3, THSD4, and VEGFC proteins (green and red; *n* = 1 technical replicate for each patient). Nuclei appear blue. White bars indicate 20 µm. Statistical analysis of gene expression in solid tumor samples was performed by one-way ANOVA with Tukey’s multiple comparison post hoc test. *p < 0.05. Astro = astrocytoma. Bar = 20 µm.

In order to verify the appearance and distribution of identified genes in a broader group of human brain tumor samples, mRNA expression of exemplary cellular dormancy-associated genes was analyzed in human glioma tissues of various malignancy grades, as well as in GBM primary cultures. With some exceptions concerning SAA2 and VEGFC expression, mRNAs of all genes were well detectable in GBM primary cultures, solid astrocytoma WHO II, III, and GBM samples (Fig. 4a, b). Interestingly, VEGFC and THSD4 were expressed significantly less in GBMs than in astrocytoma II samples. Furthermore, all proteins were abundantly found in human GBM tissues by ex vivo immunofluorescence staining (Fig. 4c). Interestingly, CCL2 and SAA2, as well as THSD4 and FSTL3 showed partial co-staining with each other, while VEGFC could only be detected in the absence of THSD4 or FSTL3. CCL2-positive cells showed pronounced co-staining with VEGFC, and partial co-staining with THSD4, but no co-staining with FSTL3. In contrast, SAA2 almost exclusively co-stained with FSTL3 and THSD4 but only partially with VEGFC.

### Identified drug-promoted cellular dormancy entry-associated and exit-associated gene expression is modulated by single chemokines in a distinct manner

**Fig. 5:**
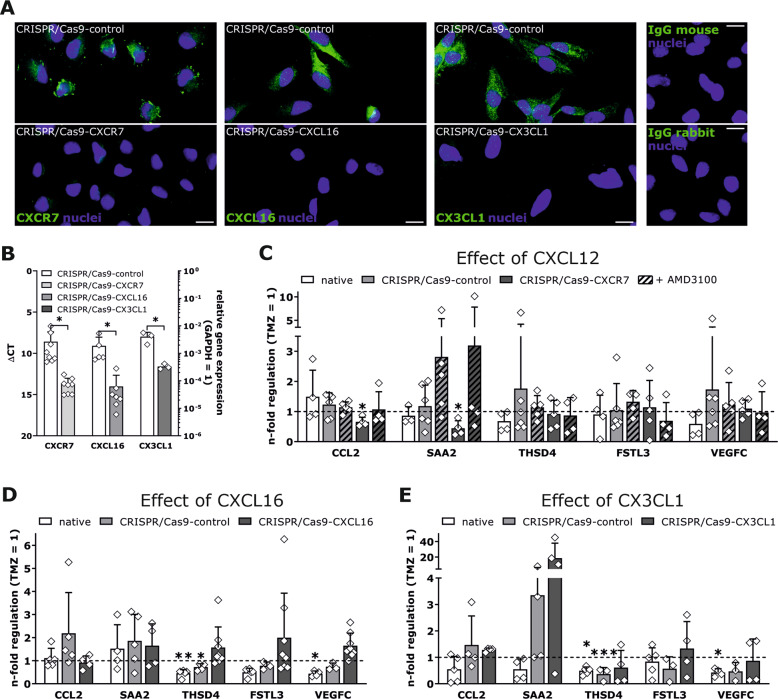
Influence of individual chemokines on gene regulation of cellular dormancy entry-associated and exit-associated genes. To assign identified gene regulations to the impact of an individual chemokine, the expression of CXCR7, CXCL16, and CX3CL1, respectively, was modified by CRISPR/Cas9 knock-out in LN229 cells. To confirm successful transfection, the CRISPR/Cas9-transfected LN229 clones were immunofluorescently stained (**a**; each with *n* = 2 biological replicates with *n* = 1 technical replicate; magnification ×200; exemplary data shown) and also analyzed by qRT-PCR (**b**; exact numbers of biological replicates for each experiment are shown in the figure; *n* = 2 technical replicates for each biological replicate). Native LN229 cells, LN229-CRISPR/Cas9 controls, as well as the corresponding CRISPR/Cas9-chemokine clones were stimulated with 500 µM TMZ or 0.5% (v/v) DMSO, respectively, with or without 2 nM of CXCL12, CXCL16, or CX3CL1 for ten days. In addition, LN229-CRISPR/Cas9-control and CRISPR/Cas9-CXCR7 cells were treated with 10 µM AMD3100 to inhibit CXCR4 signaling. Pretreated cells were incubated for a further 15 days without TMZ but with 2 nM of the respective chemokine. The gene expression of CCL2, SAA2, THSD4, FSTL3, and VEGFC was assessed by qRT-PCR after ten and 25 days. The data are shown as n-fold gene regulation with additional chemokine stimulation compared to TMZ-treatment alone (**c**–**e**; exact numbers of biological replicates for each experiment are shown in the figure; *n* = 2 technical replicates for each biological replicate). Statistical analysis of CRISPR/Cas9 transfection was performed with unpaired, two-sided Student’s *t*-test. Differences resulting from stimulation of native LN229 cells with each chemokines were analyzed by repeated one-way ANOVA with Dunnett’s multiple comparison post hoc test, and regulation in CRISPR/Cas9-transfected cells due to additional chemokine administration, and superordinate comparisons between different clones were tested by paired, two-sided Student’s *t*-test. *p < 0.05, **p < 0.01, and ***p < 0.001.

To clarify whether individual chemokines impact on drug-promoted cellular dormancy mechanisms, the influence of each chemokine alone and co-administered with TMZ during cellular dormancy entry and exit was analyzed in LN229 cells. Furthermore, chemokine signaling was abolished by CRISPR/Cas9 deletion of CXCR7, CXCL16, or CX3CL1, or by co-stimulation with AMD3100, a specific inhibitor of CXCR4 signaling [39]. Successful transfection at the mRNA and protein level is shown in Fig. 5a, b. Interestingly, stimulation of native LN229 cells with TMZ and an additional single chemokine revealed no significant reduced regulation of CCL2 or SAA2 compared with stimulation without chemokines (Fig. 5c–e, dashed line = controls with TMZ; Supplementary Fig. 2 shows controls with chemokines but without TMZ). A significantly decreased expression of CCL2 and SAA2 became visible when CXCR7-negative cells were stimulated with CXCL12 (Fig. 5c). The effects were diminished in AMD3100 co-stimulated CRISPR/Cas9-CXCR7 cells. This indicates that a balance of the CXCL12-CXCR4-CXCR7 signaling might be relevant for the regulation of cellular dormancy entry-associated genes. Since both CXCR7-negative and native LN229 cells co-stimulated with AMD3100 and TMZ showed no modulation of cellular dormancy exit-associated genes, CXCL12 stimulation had no significant effect on cellular dormancy exit (Fig. 5c). While CXCL16 and CX3CL1 showed no impact on dormancy entry in LN229 cells, both affected the changes in the expression of cellular dormancy exit-associated genes (Fig. 5d, e). Both chemokines significantly reduced the upregulation of THSD4 in control cells, and no significant change in its expression was observed after CXCL16 or CX3CL1 stimulation in CXCL16-negative or CX3CL1-negative LN229 cells. Stimulation with either CXCL16 or CX3CL1 reduced the upregulation of VEGFC expression in native, recovered LN229 cells (Fig. 5e), whereas CXCL16 or CX3CL1 knock-out cells showed no significantly altered VEGFC expression. However, this effect was only slightly detectable when compared with CRISPR/Cas9-control transfected LN229 cells. FSTL3 expression was not considerably modulated by administration of either CXCL16 or CX3CL1 in control cells, although slight inhibitory tendencies were seen (Fig. 5e).

### Effects of chemokines on dormancy entry and exit-associated genes are more complex in human GBM primary cells

**Fig. 6:**
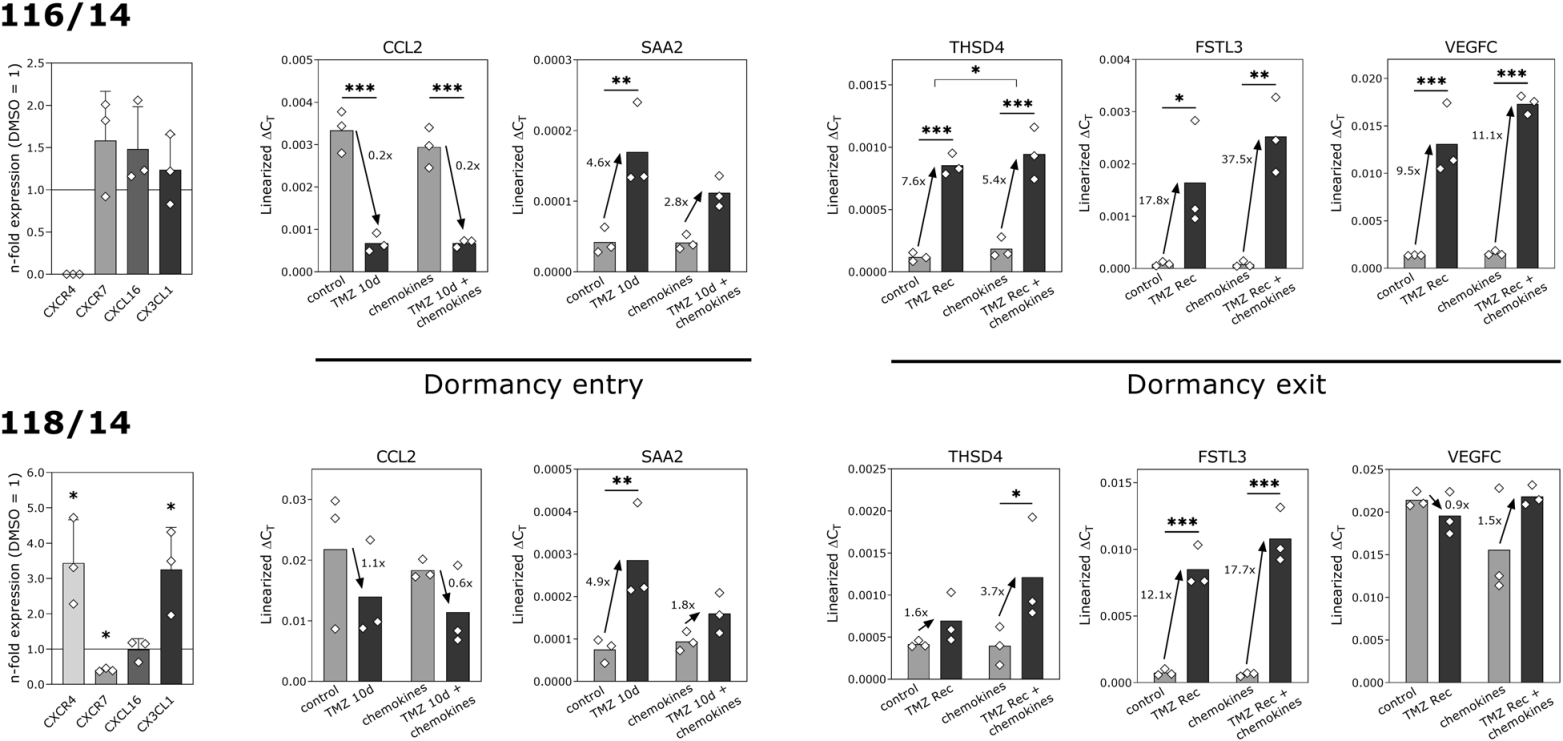
Influence of chemokines on TMZ-promoted cellular dormancy entry-associated and exit-associated gene expression in human GBM primary cultures. Human GBM primary cells 116/14 and 118/14 were treated with 500 µM TMZ or 0.5% (v/v) DMSO, respectively, with or without a chemokine mix containing 2 nM CXCL12, CXCL16, and CX3CL1 for ten days followed by a further 15 days without TMZ but with chemokine stimulation. Regulation of the respective chemokine receptors and ligands was investigated by qRT-PCR after ten days of TMZ-treatment, regulation of identified cellular dormancy entry-associated and exit-associated genes was determined by qRT-PCR after ten and 25 days of stimulation (exact numbers of biological replicates are shown in the figure, *n* = 2 technical replicates, respectively). Statistical analysis of gene regulation by TMZ-stimulation was performed by repeated two-way ANOVA with Bonferroni’s multiple comparison post hoc test. The effects with and without chemokines were analyzed by paired, two-sided Student’s *t*-test. *p < 0.05, **p < 0.01, and ***p < 0.001. Regulation of chemokine ligand and receptor gene expression was analyzed by paired, two-sided Student’s *t*-test. *p < 0.05. DMSO, dimethyl sulfoxide; Rec: recovery; TMZ, temozolomide.

Results obtained in LN229 cells were further validated using two different human primary GBM cell cultures (Fig. 6). As previously published by our group [19], these cells responded to ten days of TMZ-treatment with induction of cellular dormancy as shown by DiO-retention and higher intracellular phospho-p38 amounts in relation to phospho-p42/44 signals [19]. Furthermore, cells surviving TMZ were characterized by a negative staining for Ki-67 (Supplementary Fig. 1) and, with the exception of VEGFC for 116/14, expressed all identified cellular dormancy-associated genes (Fig. 4a). Interestingly, expression of CXCR4, CXCR7, CXCL16, and CX3CL1 was heterogeneously regulated in primary cells after ten days of TMZ-treatment (Fig. 6 left diagrams). Further, not all identified dormancy entry-associated and exit-associated genes were upregulated after TMZ-promoted cellular dormancy entry or recovery, respectively. This more complex situation was also reflected by the influence of the chemokine cocktail on the expression of the identified genes (Fig. 6). Whereas CCL2 expression was suppressed by TMZ irrespectively of additional chemokine stimulation, SAA2 upregulation could be confirmed in both GBM cells and was efficiently inhibited by the chemokine cocktail. With exception of VEGFC in 118/14 cells, all dormancy-exit associated genes were clearly upregulated in primary GBM cells after TMZ-recovery. Whereas THSD4 upregulation was decreased in 116/14 cells by additional chemokine application, VEGFC and FSTL3 expression in 116/14 cells, as well as THSD4 and FSTL3 expression in 118/14 cells was further increased by the chemokine cocktail. Thus, this cell type-specific regulation of CXCR4, CXCR7, CXCL16, and CX3CL1 in human primary GBM cells resulted in a specific influence on the TMZ-regulated expression of cellular dormancy entry-associated and exit-associated genes.

## Discussion

Dormancy has been termed one of two prerequisites for life and plays a fundamental role in all stages of tumor progression [8, 40]. Whereas in tumor mass dormancy, proliferation of tumor cells is balanced by cell death, cellular dormancy is described as a reversible quiescent cellular stage. Anti-cancer therapies promote entry into dormancy, and the local microenvironment seems to be a critical endogenous determinant for entry into and exit from dormancy [41]. In fact, chemokines as inflammatory mediators affect cancer dormancy [42–44]. Distinct chemokines, in particular CXCL12, CXCL16, and CX3CL, are essential for tumor preservation with their autocrine or paracrine signaling promoting anti-apoptotic effects or proliferation of many tumor types including GBMs [27–33].

We showed that TMZ promoted cellular dormancy in GBM cells confirming previously published results [19], while removal of TMZ led to initiation of proliferation. However, entry into cellular dormancy depended on the responsiveness of the individual glioma cell line to TMZ. Most GBM cells expressed the chemokine receptors and ligands that are necessary for signaling [27–33], and those were also significantly upregulated in TMZ-sensitive LN229 GBM cells by TMZ-stimulation.

Bruyére et al. also described a possible influence of long-term TMZ-treatment on chemokine and chemokine receptor expression [45]. Complementing these results, our model with co-administration of the chemokines CXCL12, CXCL16, and CX3CL1 resulted in LN229 GBM cells in both delayed entry and exit from TMZ-promoted cellular dormancy. This is illustrated by the influence on the expression of several genes. However, although drug-treatment induced identified dormancy-associated genes in human GBM primary cells, too, the situation in GBM primary cells seems to be more complex. Chemokines were able to regulate the expression of these genes, but in a more cell-specific manner probably caused by a more heterogeneous expression and regulation of chemokine ligands and receptors in these primary tumor cells having undergone less selection during cell culture compared to established GBM cell lines. Interestingly, the impact of cyto- and chemokines on dormancy-related pathways has been partly studied in other tumors. De Cock et al. [46] showed that inflammation triggered the escape of metastatic breast cancer cells from a dormant phase, and two other studies demonstrated that the recovery of dormant breast cancer cells depended on IL-6, IL-8, and TGF-ß1 signaling [43, 47]. Further, downregulation of CXCR4 in metastatic breast cancer cells was linked to restrained proliferation [44].

Furthermore, we showed that TMZ-promoted cellular dormancy entry-associated and exit-associated gene expression was modulated by individual chemokines in a distinct manner. The expression of the cellular dormancy entry-associated genes CCL2 and SAA2 in LN229 cells was altered by CXCL12 pointing to a relevance of the CXCL12-CXCR4-CXCR7 signaling pathways.

CCL2, also known as monocyte chemotactic protein 1 (MCP1), is a chemokine that is widely expressed in the brain, while SAA2 is an acute phase protein with cytokine-like properties that promotes neutrophil adhesion to endothelial cells during inflammation [48]. Indeed, Oh et al. [49] observed an interaction of CCL2 and CXCL12 in glioma cells. Goffart et al. [50], as well as Hattermann et al. [28] associated the CXCL12-CXCR4 signaling axis with an increased resistance of GBM stem cells, and CCL2 expression correlated with the progression of GBMs [51–54]. Here, the CCL2-CCR2 signaling axis plays a dual role in mediating early tumor immune surveillance and sustaining growth and progression of established neoplasms [55]. In addition, a senescence-associated gene signature including CCL2 was associated with a poor prognosis in human gliomas [56].

SAA2 is known to be regulated by, and is, itself able to sustain inflammatory conditions in the brain, which in turn support resistance to TMZ and induce stem cell marker expression in GBM cells in vivo [54, 57]. Since dormancy and stem cell characteristics are closely connected [19], this may support the hypothesis that SAA2 is involved in a drug-promoted cellular dormancy entry.

Interestingly, the expression of both SAA2 and CCL2 is regulated by members of the mitogen-activated protein (MAP) kinase family, especially under inflammatory conditions. As described, SAA2 transcription was induced by SAF-1, a zinc finger transcription factor that was activated by a number of inflammatory agents. SAF-1 was activated via MAP kinase-regulated phosphorylation [58]. Induction of CCL2 expression via inflammatory conditions seemed to be controlled by the interplay of c-jun N-terminal kinase (JNK), p38, and to a lesser extent of extracellular signal-regulated kinase (ERK) [59]. Since it is well known that CXCL12 activates members of the MAP kinase family in gliomas via its G-protein-coupled receptors CXCR4 and CXCR7 [22, 27, 28], it seems obvious that CXCL12 regulated CCL2 and SAA2 expression during cellular dormancy entry via these pathways.

On the other hand, CXCL16 and CX3CL1 both affected the alteration of TMZ-promoted cellular dormancy exit in LN229 cells as shown by their impact on the expression of THSD4 and, to some extent, of VEGFC. However, significant FSTL3 regulation required stimulation with all three chemokines.

Indeed, to escape angiogenic dormancy, the tumor undergoes an angiogenic switch leading to enhanced release of proangiogenic factors [9]. Besides its proangiogenic function, VEGFA promoted tumor dormancy reversal [52]. Thus, it can be speculated that VEGFC plays a similar role. Interestingly, CX3CL1 is a regulator of angiogenesis, and stimulation of macrophages with CX3CL1 resulted in a reduced VEGFA expression [60]. VEGFC expression is regulated via the PI3K-AKT-mTOR signaling pathway [61]. Since CXCL16 was able to activate Akt via inverse signaling mechanisms in brain tumors [62], the PI3K-AKT signaling pathway might be involved in the regulation of VEGFC expression via CXCL16, and probably via CX3CL1, during cellular dormancy exit.

Concerning the role of THSD4 in cellular dormancy exit, repression of thrombospondin-1 activity induced angiogenesis and proliferation in persistent tumors [63]. THSD4 is a protease, also known as an ADAM with thrombospondin motifs-like protein 6 (ADAMTSL6) that was upregulated during GBM recovery. However, other ADAMs with thrombospondin motifs (ADAMTS4, ADAMTS5) positively impacted the proliferation of glioma cells in vitro and potentially promoted their invasion by cleavage of brevican, a major component of brain tissue [64]. Cohen et al. [65] demonstrated that THSD4 expression was regulated by the GATA binding protein 3 (GATA3). This protein belongs to a family of tissue specific transcription factors and is able to transactivate its target genes [65]. Chemokines were able to inhibit the expression of GATA3 [66]. Thus, a reduction of GATA3 expression may be involved in CXCL16-regulated or CX3CL1-regulated inhibition of THSD4 expression in gliomas.

Apart from this, upregulation of FSTL1, a homolog of FSTL3, increased the TMZ resistance of GBM cells [67] and was correlated with proliferation and colony formation [68]. FSTL3 is a secreted glycoprotein involved in the regulation of various biological effects through its binding to members of the TGFβ superfamily. TNFα has been shown to activate FSTL3 expression at the transcriptional level through a responsive element which binds the transcription factor nuclear factor κB (NF-κB) [69]. Furthermore, TGFβ promoted the effect of TNFα on FSTL3 expression [69]. Since the kinase Akt regulates the transcriptional activity of NF-κB [70], and chemokines are able to regulate Akt [62], Akt-NF-κB-mediated signaling is probably involved in the regulation of FSTL3 expression by chemokines during TMZ-promoted cellular dormancy exit.

In conclusion, we demonstrated that chemokines play a significant role in the regulation of drug-promoted cellular dormancy entry and exit pointing to a fine-tuning influence of these molecules on distinct cellular dormancy processes in glioblastomas.

## Materials and methods

### Human specimens

Freshly dissected glioma samples of various malignancy grades were obtained at the Department of Neurosurgery (Kiel, Germany) in accordance with the Helsinki Declaration of 1975 and with approval of the ethics committee of the University of Kiel, Germany, after written informed consent of the donors (file references: D471/15 and D524/17). The diagnosis was made by the Department of Pathology (UKSH, Kiel, Germany).

### Cultivation of GBM cell lines and human primary GBM cells

The human glioblastoma cell lines T98G (ECACC No. 92090213) and LN229 (ATCC-CRL-2611) were obtained from the European Collection of Cell Cultures (ECACC, Salisbury, UK) or the American Type Culture Collection (ATCC, Manassas, Virginia, USA) and cultured as described previously [32]. Human primary GBM cultures were produced by dissociation and cultured according to established techniques as previously described [32]. Purity of the GBM cells was ascertained by immunostaining with cell type-specific markers, and by the absence of contamination with mycoplasms, and GBM cell line identity was verified by short tandem repeat profiling as previously described [19].

### Stimulation of GBM cells

1.5 × 10^5^ T98G, LN229, 116/14, or 118/14 cells, respectively, were stimulated for ten days with 500 µM TMZ (Sigma-Aldrich, St. Louis, MO, USA) dissolved in dimethyl sulfoxide (DMSO, Merck, Darmstadt, Germany) in Dulbecco’s modified Eagle’s medium (DMEM; Thermo Fisher Scientific, Waltham, MA, USA) supplemented with 10% fetal bovine serum (FBS; Thermo Fisher Scientific). DMSO 0.5% (v/v) was used as control. Medium was changed, and the cells were cultured for a further 15 days without TMZ-stimulation. In addition, 2 nM CXCL12, CXCL16, and CX3CL1 (recombinant human proteins, PeproTech GmbH, Hamburg, Germany) were added individually or as chemokine cocktail. Stimulation with the individual chemokines and with the cocktail was continued during the 15-day period following TMZ-treatment. These experiments were analyzed by reverse transcription and quantitative real-time polymerase chain reaction (qRT-PCR), western blot, and immunocytochemistry after ten days of stimulation, as well as after further 15 days without TMZ-stimulation as described below. The effects of the individual chemokines were proven using LN229 cells with CRISPR/Cas9 knock-out of CXCR7, CXCL16, or CX3CL1, respectively, and in which CXCR4 signaling was specifically inhibited by addition of 10 μM AMD3100 (selective CXCR4 inhibitor, Merck). To prove TMZ-promoted cellular dormancy and recovery, the cells were stained with Vybrant® DiO cell-labeling solution (Thermo Fisher Scientific) according to manufacturer’s instructions, and dye retention was monitored at day 10, as well as at days 7 and 15 after TMZ-stimulation by transmitted light and fluorescence microscopy (AxioObserver.Z1; Carl Zeiss, Oberkochen, Germany) with equal exposure times in relation to controls using the ZEN2 software. Finally, stained and vital cells were counted in every section from each experiment in at least eight different fields of view at 100-fold magnification, and the ratio of stained cells to total number of vital cells was calculated.

### Microarray-based transcriptome analysis

RNA of differentially treated LN229 cells was isolated with the TRIzol® reagent (Thermo Fisher Scientific). All following steps including microarray-based transcriptome analysis were performed by OakLabs GmbH (Hennigsdorf, Germany) using the ArrayXS® human 8 × 60 K microarray (Agilent Technologies, Santa Clara, CA, USA) that covers 59,508 coding and non-coding genes. In detail, the integrity of RNA samples was assessed using the Bioanalyzer’s RNA integrity number (RIN) to determine the quality and quantity of the samples. For labeling, the low input QuickAmp® labeling kit (Agilent Technologies) was used to generate fluorescent cRNA, and an in vitro transcription was performed to obtain cRNA labeled with cyanine 3-CTP. After hybridization with the Agilent gene expression hybridization kit (Agilent Technologies), fluorescence signals were detected by the SureScan® microarray scanner (Agilent Technologies). Background subtracted signals were quantile normalized using the ranked mean quantiles, and n-fold expression differences were presented after logarithmic base 2 transformation, i.e., log2 (expression ratio) = log2-fold change (log2FC). Negative and positive log2FC values indicate overexpression and downregulation, respectively, in relation to controls, e.g., a log2FC = 2 indicates a 4-fold expression difference. Statistical analysis was performed using Welch’s *t*-test. Log2FC values between −1 and 1 were not considered, and only statistically significant values (*p* < 0.05) were displayed. Venn diagrams were prepared to visualize the influence of chemokines on TMZ-promoted cellular dormancy entry and exit. Complete gene lists including log2FC and *p* values of different Venn areas are given in Supplementary Tables 1 and 2.

### CRISPR/Cas9 gene knock-out

LN229 cells were seeded into a six-well plate (1.5 × 10^5^ cells/well), overlaid with DMEM containing 10% FBS, and the CRISPR/Cas9 plasmid solution (Santa Cruz Biotechnology, Dallas, TX, USA) was added drop-wise according to the manufacturer’s instructions. Transfection was performed overnight at 37 °C. To ensure gene knock-out, successfully transfected clones were selected by their acquired resistance to puromycin [1 μg/ml puromycin (InvivoGen, San Diego, CA, USA)]. Individual clones were picked, and knock-out efficiency was analyzed by qRT-PCR and immunocytochemistry as described below.

### Reverse transcription and quantitative real-time PCR (qRT-PCR)

RNAs of tissues and cells were isolated with the TRIzol® reagent (Thermo Fisher Scientific) or with the ARCTURUS® PicoPure® RNA isolation kit (Applied Biosystems, Waltham, MA, USA) according to the manufacturer’s instructions. DNase digestion, cDNA synthesis, and qRT-PCR were performed as described previously [19, 33] using TaqMan primer probes (Applied Biosystems) listed in Supplementary Table 3. Cycles of threshold (C_T_) were determined, and the ∆C_T_ values of each sample were calculated as CT_gene of interest_ – CT_GAPDH_. ∆C_T_ values or, for better comparison, linearized ∆CT values are shown in the figures. The induction of gene expression after stimulation is displayed as n-fold expression changes = 2^∆CT control − ∆CT stimulus^.

### Immunohistochemistry and immunocytochemistry

Cryostat sections of GBM tissues and glass cover slips of differentially treated cells were prepared as described previously [19, 32]. Cells were incubated overnight with the primary antibodies at 4 °C, followed by the secondary antibodies for 1 h at 37 °C. The nuclei were counterstained with 4′,6-diamidino-2-phenylindole, and the embedded slides were analyzed using light and fluorescence microscopy (AxioObserver.Z1; Carl Zeiss) with equal exposure times in relation to controls using the ZEN2 software. Used primary antibodies are listed in Supplementary Table 4. If primary antibodies were derived from the same species, non-specific binding was blocked by F(ab) fragments derived from that species (1:1000, from Jackson ImmunoResearch, West Grove, PA, USA). Primary antibodies were omitted for negative controls. For secondary antibody controls, IgG mouse (MAB002; R&D Systems, Minneapolis, MN, USA) or IgG rabbit (AB-105-C; R&D Systems) control antibodies were used at the same concentrations as the replaced primary antibodies. Donkey anti-mouse or anti-rabbit IgGs labeled with Alexa Fluor 488 or Alexa Fluor 555 (1:1,000; Thermo Fisher Scientific) served as secondary antibodies.

### Western blot

Differentially treated cells were harvested, and 3 to 30 µg of protein per sample were used for western blotting as described previously [32]. Used primary antibodies are listed in Supplementary Table 5. Secondary antibodies were donkey anti-rabbit, anti-mouse or anti-goat IgG-HRP (1:40,000, 1:30,000 or 1:100,000; Santa Cruz Biotechnology). As loading control of equal amount of protein, GAPDH was detected as described previously [32]. Signal densities were measured using ImageJ® software. For phosphorylated kinases the signals were normalized to GAPDH, and phospho-p38/phospho-p42/44 ratios were calculated for DMSO-treated and TMZ-treated samples; for CCL2, FSTL3, THSD4, SAA2, and VEGFC the signals were normalized to GAPDH, and the n-fold signal induction was determined in relation to respective control samples (control/chemokine = 1).

### Statistical analysis

The data were statistically analyzed using the GraphPad Prism 8.4® software (GraphPad Software, San Diego, CA, USA). To ensure an adequate power to detect a pre-specified effect size, all experiments were performed whenever applicable with a minimum of *n* = 3 independent biological replicates. Samples were only included in the study if they met established internal quality control criteria, i.e., no mycoplasm contamination, correct short tandem repeat profiling, correct histological diagnosis etc. Depending on the experimental setup either a Student *t*-test, a one-way analysis of variance (ANOVA) or a two-way ANOVA was performed, as indicated for each experiment in the individual figure legends. The data fulfilled the preconditions of the tests, and the variance between the statistically compared groups was similar. The samples sizes and a description of the sample collection including the number of biological/technical replicates are exactly described in the figure legends. In general, the data are presented as mean ± standard deviation (SD). Statistical significance is marked with asterisks depending on the *p* value: *p < 0.05, **p < 0.01, and ***p < 0.001.

## Supplementary information

Supplementary figures and tables legends

Supplementary figure 1

Supplementary figure 2

Supplementary table 1

Supplementary table 2

Supplementary table 3

Supplementary table 4

Supplementary table 5
