# Supplementary material for: Entry and exit of chemotherapeutically-promoted cellular dormancy in glioblastoma cells is differentially affected by the chemokines CXCL12, CXCL16, and CX3CL1
Source: Oncogene. 2020 Apr 28;39(22):4421–35. doi: 10.1038/s41388-020-1302-8 (PMC7253351; doi:10.1038/s41388-020-1302-8)
Supplement: Supplementary file 1 — Supplementary figures and tables legends [file 41388_2020_1302_MOESM1_ESM.docx]

**Entry and exit of chemotherapeutically-promoted cellular dormancy in glioblastoma cells is differentially affected by the chemokines CXCL12, CXCL16 and CX3CL1**

Vivian Adamski^1^, Kirsten Hattermann^2^, Carolin Kubelt^1^, Gesa Cohrs^1^, Ralph Lucius^2^, Michael Synowitz^1^, Susanne Sebens^3^, Janka Held-Feindt^1^

*^1^Department of Neurosurgery, University Medical Center Schleswig-Holstein UKSH, Campus Kiel, 24105 Kiel, Germany; ^2^Department of Anatomy, University of Kiel, 24118 Kiel, Germany; ^3^Institute for Experimental Cancer Research, University of Kiel and University Medical Center Schleswig-Holstein UKSH, Campus Kiel, 24105 Kiel, Germany*

# Correspondence to:

# Prof. Dr. Dr. Janka Held-Feindt, PhD, MD

Department of Neurosurgery, University Medical Center of Schleswig-Holstein, Campus Kiel, Arnold-Heller-Str. 3, Building 41, 24105 Kiel, Germany

Phone: +49(0)431-500-23679, Fax: +49(0)431-500-23678

Email: Janka.Held-Feindt@uksh.de

**Running title:** Chemokine influence on dormancy entry and exit

**Conflict of interest:** The authors declare no conflicts of interests.

**Text summary for:**

**Supplementary tables 1 and 2:** Gene lists of microarray analysis on cellular dormancy entry and exit including log2FC and p values and listed according to Figure 2 (n= 3 biological replicates). Expression differences were displayed as log2-fold changes (log2FC) with a log2FC=2 value indicating a 4-fold expression difference.

**Supplementary table 3:** TaqMan primer probes (Applied Biosystems, Waltham, MA, USA) used in the study.

**Supplementary table 4:** Primary antibodies used for immunohistochemical, immunocytochemical and immunofluorescence staining in the study.

**Supplementary table 5:** Primary antibodies used for western blotting in the study.

**Supplementary Figure 1:** Ki-67 fluorescence-staining of different glioblastoma cells. Partially drug-sensitive LN229, 116/14 and 118/14 cells were stimulated with 500 µM TMZ or DMSO, respectively, for ten days, and Ki-67 fluorescence-staining was performed (n=2 biological replicates with n=1 technical replicate, respectively; exemplary data shown). DMSO, dimethyl sulfoxide; TMZ, temozolomide. Bar = 20µm.

**Supplementary Figure 2:** Influence of chemokines on gene regulation of cellular dormancy-associated genes. LN229 cells, LN229-CRISPR/Cas9 controls and CRISPR/Cas9-chemokine clones were stimulated with/without chemokines for ten and 25 days, respectively. Statistical analysis was performed by paired, two-sided Student’s t-test, repeated one-way ANOVA with Dunnett’s or Sidak’s multiple comparison post hoc test. *p<0.05.
