# Supplementary figures and images for: Entry and exit of chemotherapeutically-promoted cellular dormancy in glioblastoma cells is differentially affected by the chemokines CXCL12, CXCL16, and CX3CL1
Source: Oncogene. 2020 Apr 28;39(22):4421–35. doi: 10.1038/s41388-020-1302-8 (PMC7253351; doi:10.1038/s41388-020-1302-8)

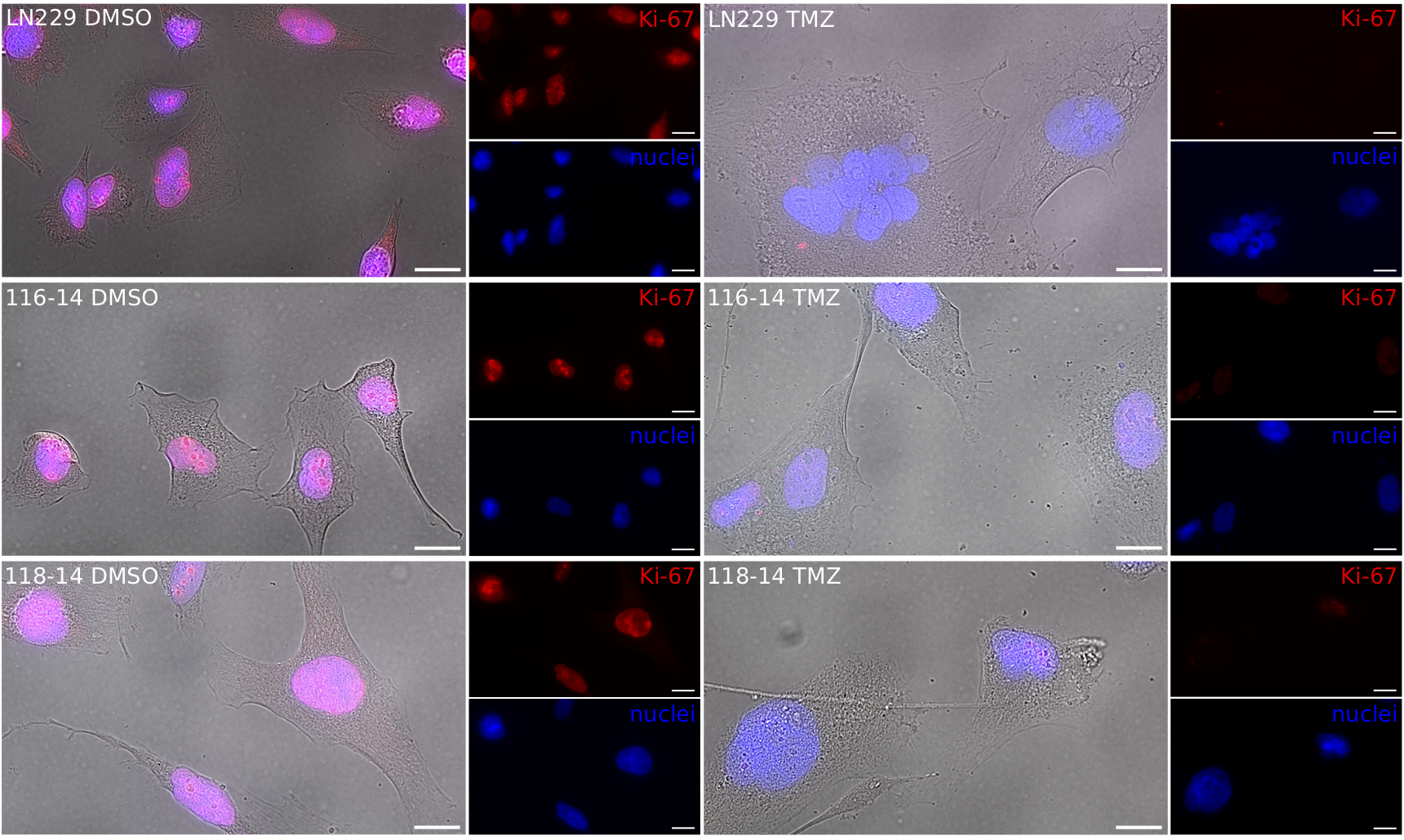

Supplement: Supplementary file 2 — Supplementary figure 1 [file 41388_2020_1302_MOESM2_ESM.tif]

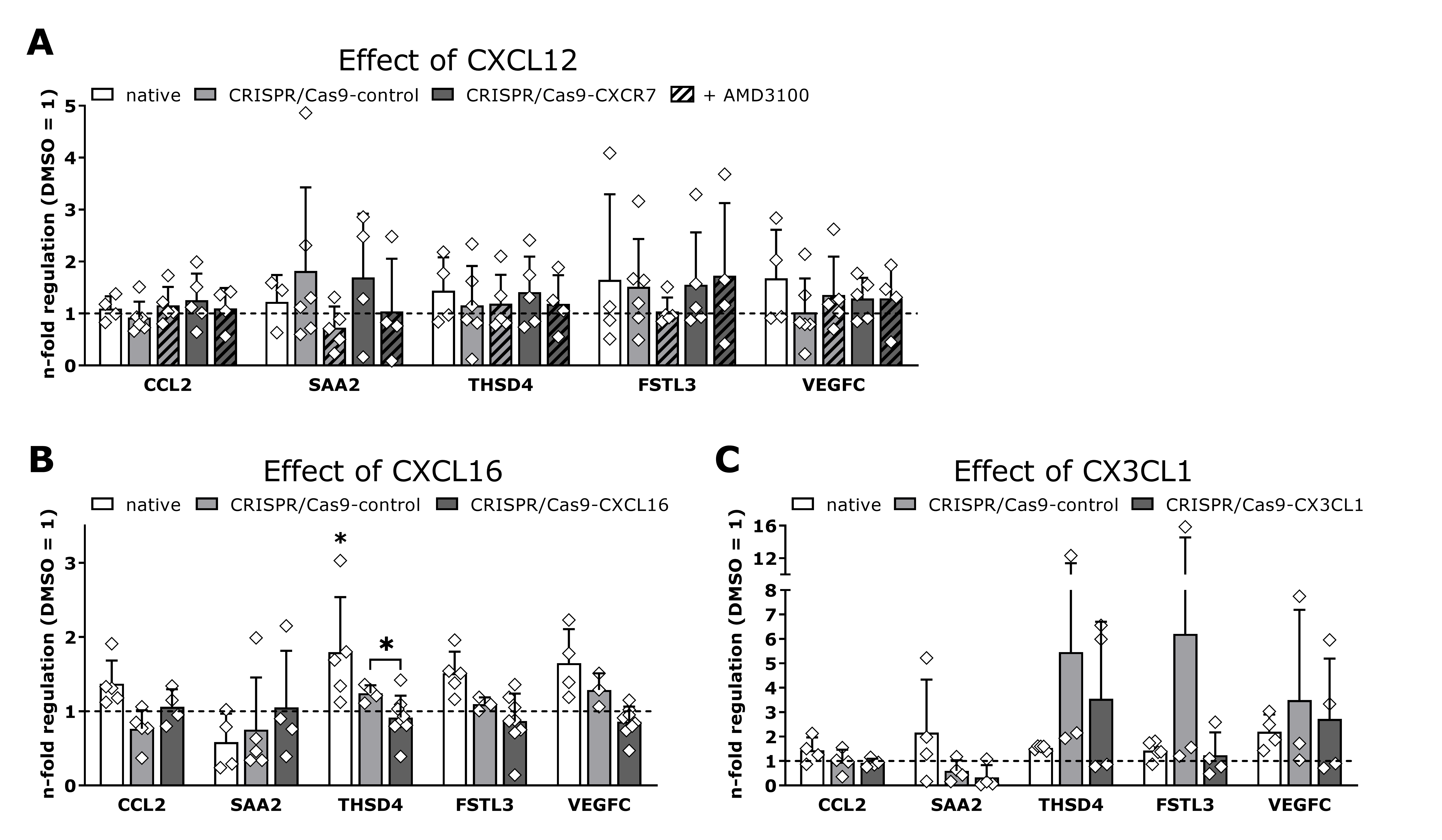

Supplement: Supplementary file 3 — Supplementary figure 2 [file 41388_2020_1302_MOESM3_ESM.tif]
