# Supplementary material for: Entry and exit of chemotherapeutically-promoted cellular dormancy in glioblastoma cells is differentially affected by the chemokines CXCL12, CXCL16, and CX3CL1
Source: Oncogene. 2020 Apr 28;39(22):4421–35. doi: 10.1038/s41388-020-1302-8 (PMC7253351; doi:10.1038/s41388-020-1302-8)
Supplement: Supplementary file 6 — Supplementary table 3 [file 41388_2020_1302_MOESM6_ESM.docx]

**Supplementary table 3:** TaqMan primer probes (Applied Biosystems, Waltham, MA, USA) used in the study.

| **Gene** | **Primer** |
| --- | --- |
| CCL2 | Hs00234140_m1 |
| CX3CL1 | Hs00171086_m1 |
| CXCL16 | Hs00222859_m1 |
| CXCR4 | Hs00237052_m1 |
| CXCR7 | Hs00664172_m1 |
| Glycerinaldehyde 3-phosphate dehydrogenase (GAPDH) | Hs99999905_m1 |
| Follistatin-related protein 3 (FSTL3) | Hs00610505_m1 |
| Serum amyloid A protein 2 (SAA2) | Hs01667582_m1 |
| Thrombospondin type 1 domain containing 4 (THSD4) | Hs00388227_m1 |
| Vascular endothelial growth factor C (VEGFC) | Hs01099203_m1 |
