# Supplementary material for: Entry and exit of chemotherapeutically-promoted cellular dormancy in glioblastoma cells is differentially affected by the chemokines CXCL12, CXCL16, and CX3CL1
Source: Oncogene. 2020 Apr 28;39(22):4421–35. doi: 10.1038/s41388-020-1302-8 (PMC7253351; doi:10.1038/s41388-020-1302-8)
Supplement: Supplementary file 7 — Supplementary table 4 [file 41388_2020_1302_MOESM7_ESM.docx]

**Supplementary table 4:** Primary antibodies used for immunohistochemical, immunocytochemical and immunofluorescence staining in the study.

| **Antibody** | **Dilution** | **catalog no.** | **company** |
| --- | --- | --- | --- |
| Mouse anti-**CCL2** | 1:500 | MA5-17040 | Thermo Fisher Scientific, Waltham, MA, USA |
| Mouse anti-**CX3CL1** | 1:200 | MAB3651 | R&D Systems,  Minneapolis, MN, USA |
| Rabbit anti-**CXCL16** | 1:100 | 500-P200 | PeproTech GmbH,  Hamburg, Germany |
| Mouse anti-**CXCR7** | 1:100 | MAB42273 | R&D Systems,  Minneapolis, MN, USA |
| Rabbit anti-**FSTL3** | 1:100 | AP12300b | Abgent,  San Diego, CA, USA |
| Rabbit anti-**SAA2** | 1:200 | 13192-1-AP | Proteintech Europe, Manchester, UK |
| Rabbit anti-**THSD4** | 1:500 | 20619-1-AP | Proteintech Europe,  Manchester, UK |
| Mouse anti-**VEGFC** | 1:200 | ab106512 | Abcam,  Cambridge, UK |
