# Supplementary material for: Entry and exit of chemotherapeutically-promoted cellular dormancy in glioblastoma cells is differentially affected by the chemokines CXCL12, CXCL16, and CX3CL1
Source: Oncogene. 2020 Apr 28;39(22):4421–35. doi: 10.1038/s41388-020-1302-8 (PMC7253351; doi:10.1038/s41388-020-1302-8)
Supplement: Supplementary file 8 — Supplementary table 5 [file 41388_2020_1302_MOESM8_ESM.docx]

**Supplementary table 5:** Primary antibodies used for western blotting in the study.

| **Antibody** | **Dilution** | **catalog no.** | **company** |
| --- | --- | --- | --- |
| Rabbit  anti-**GAPDH** | 1:200 | sc-47724 | Santa Cruz Biotechnology,  Heidelberg, Germany |
| Rabbit  anti-**phospho-p42/44** | 1:1,000 | #9101 | Cell Signaling,  Danvers, MA, USA |
| Rabbit  anti-**phospho-p38** | 1:100 | #4511 | Cell Signaling,  Danvers, MA, USA |
| Mouse  anti-**CCL2** | 1:500 | MA5-17040 | Thermo Fisher Scientific,  Waltham, MA, USA |
| Goat  anti-**FSTL3** | 1:120 | PA5-47106 | Thermo Fisher Scientific,  Waltham, MA, USA |
| Rabbit  anti-**SAA2** | 1:200 | 13192-1-AP | Proteintech Europe, Manchester, UK |
| Rabbit  anti-**THSD4** | 1:500 | 20619-1-AP | Proteintech Europe, Manchester, UK |
| Mouse  anti-**VEGFC** | 1:200 | ab106512 | Abcam,  Cambridge, UK |
